# Editorial on common mental health disorders and cognitive decline in a longitudinal Down syndrome cohort

**DOI:** 10.1192/bjo.2024.16

**Published:** 2024-04-11

**Authors:** Samuel J. Tromans, Pushpal Desarkar

**Affiliations:** Department of Population Health Sciences, University of Leicester, Leicester, UK; and Psychiatry of Intellectual Disability at Leicestershire Partnership NHS Trust, Leicester, UK; Department of Psychiatry, University of Toronto, Canada; and Adult Neurodevelopmental Services, Centre for Addiction of Mental Health, Toronto, Canada

**Keywords:** Down syndrome, intellectual disability, dementia, anxiety, depression

## Abstract

This editorial discusses a study by Idris and colleagues, where the authors investigated the impact of common mental disorders (CMDs) among patients with Down syndrome, with respect to development of clinical features of Alzheimer's disease.

Pushpal Desarkar is a member of the International Editorial Board for BJPsych Open. He is currently being supported by the Canadian Institutes of Health Research (CIHR), Innovation Fund from the Alternate Funding Plan of the Academic Health Sciences Centres of Ontario, CAMH Discovery Fund, and the Academic Scholar Award from the Department of Psychiatry, University of Toronto.

Down syndrome (referred to as trisomy 21 in the 11th edition of the International Classification of Diseases^1^), is a ‘chromosomal abnormality, characterised by the presence of a third (partial or total) copy of chromosome 21, with multiple associated clinical manifestations, including variable intellectual disability, and an increased risk of multiple pathologies, including cardiac and gastrointestinal abnormalities, epilepsy, endocrine conditions (e.g. hypothyroidism) and Alzheimer's disease. In the UK, Down syndrome has a prevalence of around 0.07% in men and around 0.06% in women.^2^ Additionally, people with Down syndrome have a higher prevalence of a variety of forms of mental illness relative to the general population, including anxiety, and affective and psychotic disorders.^3^

People with Down syndrome are at substantially heightened risk of Alzheimer's disease, with almost all individuals having Alzheimer's disease pathology evident by the age of 40 years.^4^ Developing a greater understanding of Alzheimer's disease in Down syndrome is essential in improving the care of the Down syndrome community, but may also inform treatment of Alzheimer's disease within the general population.^4^ In their study, Idris and colleagues^5^ investigate common mental disorders (CMDs) in patients with Down syndrome, and whether they are associated with subsequent development of features of Alzheimer's disease.

Recruitment for this study benefited from working with an established cohort of patients with Down syndrome from the London Down Syndrome Consortium Study. Such pre-established large longitudinal cohorts can enable recruitment of sizeable study populations for even relatively uncommon studies, helping ensure that the subsequent findings are more likely to have meaningful clinical, research and policy implications.^6^ Study populations can be further increased in size through international collaboration across multiple research groups and respective associated cohort groups. Furthermore, this study also focuses on a research priority reported by a collaboration of Down syndrome experts and charities,^7^ charting the trajectories of cognitive functioning in patients through the use of longitudinal cohorts.

Another benefit of this approach was that participants have pre-determined baseline levels of cognitive function, providing a patient-specific means of comparison with subsequent findings on neuropsychological testing. Indeed, the authors advocate for baseline cognitive assessment for all people with Down syndrome, to provide a point of comparison in the event of future cognitive decline. Such a pre-emptive approach would require forethought and planning, but would be of considerable benefit to the Down syndrome community. Otherwise, there is a risk that individuals with Down syndrome, particularly those with no prior history of mental illness, only receive specialised input and related cognitive assessment from specialist learning disability services after they have developed significant Alzheimer's disease-like symptoms, leading to a referral, often from primary care.

The study design adopts a thorough approach to assessment of CMDs among patients with Down syndrome, incorporating report of CMD symptoms by each individual's doctor, psychotropic medication history, and extensive caregiver interview and questionnaire assessment. Such a multi-pronged approach can be invaluable when assessing for presence of mental health in people where many may have impairments in verbal communication, and thus clinical observation skills and informant history are invaluable in providing further clinical information.^8^ There are widely used and validated CMD interview measures for the general population, such as the revised Clinical Interview Schedule (CIS-R),^9^ but unfortunately this measure is not validated for patients with intellectual disability, and thus could not be used in a study of this type. As an alternative to a validated interview-based measure, the CMD identification process could have been further strengthened by the clinically qualified members of the research team undertaking clinical assessments of all study participants, particularly if such assessments were carried out by intellectual disability specialist clinicians, who would be experienced in interpreting non-verbal clinical signs among patients who often present with substantial communication impairment. In addition to the CMD-related tests, participants underwent a comprehensive battery of cognitive assessments that were suited to individuals with a range of abilities, which is essential when evaluating cognition in people with intellectual disabilities, where not all assessments used in the general population will be valid for use.

Furthermore, including study participants lacking capacity where possible is important in research involving groups of patients where learning disability is prevalent, as otherwise there is a risk of potentially disproportionately excluding people with greater levels of intellectual impairment. This can have the unintended consequences of making the study population less representative of the Down syndrome community, but also may prevent obtaining important research data on those individuals with the greatest levels of intellectual impairment, where such information is essential to inform best practice. As Iacona^10^ writes, there are ethical challenges with respect to including patients in research who are unable to consent for themselves, with a need to balance the potential benefits of taking part in research (both to participants and the wider Down syndrome community) and the rights of people with intellectual disability to take part in research while being mindful of the potential harms. The authors report their inclusion of patients with severe intellectual disability as a strength of the study, and also describe a process of carer involvement in decisions as to whether potential participants lacking capacity to consent could still take part in the study.

The authors clearly define what they mean by CMDs in the context of this study, defining them as ‘a diagnosis of depression, anxiety, or obsessive-compulsive disorder, including seasonal affective disorder (SAD), post-traumatic stress disorder (PTSD), and disruptive behaviour disorders’, and clearly justify their rationale for the study: that such disorders are associated with Alzheimer's disease-like cognitive deterioration in the general (non-Down syndrome) population. Indeed, meta-analyses report both depression^11^ and anxiety^12^ as risk factors for subsequent development of Alzheimer's disease within the general population.

At baseline, 27 of the 115 study participants (23%) had an identified CMD. Mantry et al^13^ previously reported prevalence of mental ill health of any type among a cohort of patients with Down syndrome (*n =* 186), reporting rates ranging from 23.7% from clinical assessment to 10.8% for Diagnostic and Statistical Manual of Mental Disorders, Fourth Edition Revised (DSM-IV-TR) criteria.^14^ As the authors defined mental ill health as a far broader concept than CMD, since it also encompassed conditions such as psychotic disorders, substance abuse disorders, autism and attention deficit hyperactivity disorder, it seems surprising that the CMD prevalence was similar to the highest prevalence figure reported by Mantry et al.^13^ However, this may be in part a reflection of the size of the respective study populations; there is a lack of large-scale epidemiological studies on mental ill health prevalence among people with Down syndrome, and international collaboration between specialist centres is likely required to achieve such clinically valuable data.

The study did not find any association between presence of stable and/or treated CMDs and cognitive decline over a 3-year period. The authors explained this could be in part due to 50% of the participants receiving pharmacological treatment, which potentially could have mitigated the risk. However, there is a need for future research to clarify any potential neuroprotective effect of antidepressant treatment in Down syndrome (as research involving animal models has reported hippocampal neurogenesis in response to chronic antidepressant treatment^15^), or whether such effects are entirely attributable to an improvement in CMD-related symptomatology. It is also possible that the longitudinal course of cognitive decline in Down syndrome is independent of CMDs, particularly if they are well managed. One take-home point for clinicians is that the diagnostic possibility of Alzheimer's disease should be considered when typical symptoms are present, irrespective of the presence of CMDs.

The authors correctly point out the limitation of using receipt of selective serotonin reuptake inhibitor (SSRI) and serotonin-noradrenaline reuptake inhibitor (SNRI) prescriptions as a proxy indicator of CMD, in that these medications can be prescribed for other non-CMD-related indications. Additionally, such prescriptions may have originally been commenced for CMDs that have since resolved, and may not recur were the medications to be discontinued. This makes it difficult to distinguish between patients with ongoing CMDs receiving effective treatment, and patients with a history of CMDs who happen to be still receiving SSRI or SNRI treatment.

## Conclusion

This study found that stable and/or treated CMDs were not associated with progressive Alzheimer's disease-like deterioration in cognition and functioning. The authors suggest that such findings indicate the importance of baseline cognitive assessment in all people with Down syndrome prior to dementia onset. This study provides valuable insights into the relationship between a CMD diagnosis and longer-term cognitive outcomes in individuals with Down syndrome.

## Data Availability

Data availability is not applicable to this article as no new data were created or analysed in this editorial.
